# Evaluation of Side-to-Side Differences in Lower Extremity Sensory Nerve Action Potential (SNAP) Amplitude in Relation to Motor Nerve Conduction Studies

**DOI:** 10.7759/cureus.94961

**Published:** 2025-10-20

**Authors:** Handan Uzunçakmak-Uyanık, Merve Melodi Çakar, Refah Sayın

**Affiliations:** 1 Neurology, Ufuk University, Ankara, TUR; 2 Neuro-Electrophysiology, Institute of Neurological Sciences and Psychiatry, Hacettepe University, Ankara, TUR; 3 Neurology, Hacettepe University, Ankara, TUR

**Keywords:** anatomical variation, asymmetry, side-to-side amplitude difference, superficial peroneal sensory nerve, sural nerve

## Abstract

Introduction and aim: Anatomical variations and technical factors can lead to side-to-side differences in nerve conduction studies, potentially causing false positive diagnoses. This study aimed to define normal limits of side-to-side differences in sural and superficial peroneal (SP) sensory nerve action potential (SNAP) amplitudes, considering their association with lower extremity motor nerves.

Methods: Fifty healthy adults were assessed for lower extremity compound muscle action potential (CMAP) and SNAP amplitudes. The upper limit of side-to-side differences was defined as the mean plus two standard deviations. Associations between CMAP and SNAP amplitudes, based on motor-sensory nerve relationships, were analyzed using Pearson correlation and linear regression. CMAP amplitude differences were used as the references.

Results: Significant positive correlations were found between left peroneal CMAP and sural SNAP (r=0.451, p=0.001), and between right peroneal CMAP and SP SNAP (r=0.304, p=0.032). Peroneal CMAP amplitude significantly affected left sural and right SP SNAP amplitudes (B=1.246, p=0.001; B=0.672, p=0.032). If the peroneal CMAP side-to-side difference is ≤4.04 mV, sural and SP SNAP differences up to 7.76 and 6.7 µV, respectively, may be considered normal.

Conclusions: Considering peroneal CMAP amplitude asymmetry may help improve the interpretation of lower extremity SNAP differences and reduce the risk of false positives.

## Introduction

Sural and superficial peroneal (SP) sensory nerve conduction studies (NCSs) are of value in the electrophysiological diagnosis of polyneuropathy [1]. These NCSs are also critical for diagnosing lumbosacral plexopathy and mononeuropathies of the sciatic, tibial, and peroneal nerves [2]. In general, a side-to-side sensory nerve action potential (SNAP) amplitude difference of up to 50% is considered normal in many centers. This is due to factors such as stimulation and recording techniques, the effects of temporal dispersion with increasing conduction distance [3], and anatomical variations [4,5]. On the other hand, we know that the effect of temporal dispersion on amplitude and shape is dependent on the number and distribution of conducting fibers, and that changes with fiber loss or increased conduction velocity variability in SNAP may be smaller than expected [3]. This highlights the need for more sensitive criteria when evaluating side-to-side SNAP differences. Moreover, excessive side-to-side differences in sensory nerve amplitudes between extremities may lead to false-positive electrophysiological diagnoses. Taking the association with lower extremity motor nerves into account, this study aimed to determine the cut-off values ​​for amplitude differences of the sural and SP nerves between the two lower extremities that could be regarded as within the normal range.

## Materials and methods

Participants

Following approval from the local ethics committee (#61351342/020-157), 50 healthy adult volunteers aged 20 to 69 years were recruited over six months at the EMG Unit of Ufuk University, after obtaining informed consent. All participants were confirmed to be neurologically healthy and free of systemic diseases through clinical interviews and examinations. Age, height, and body mass index were recorded for each participant.

Electrophysiology

Bilateral tibial and peroneal motor, as well as sural and SP sensory nerve conduction studies (NCSs), were performed using a Medelec Synergy EMG machine (Old Woking, United Kingdom: Oxford Instruments Medical Ltd.). Skin temperature in both extremities was kept ≥32°C and the same. Surface cup electrodes were used as the active, reference, and ground electrodes. Filter settings were 20 Hz to 2 kHz for sensory NCSs and 20 Hz to 20 kHz for motor NCSs.

Participants were examined in the supine position. Motor NCSs were performed orthodromically, with compound motor action potentials (CMAPs) recorded using a tendon-belly montage. For tibial motor studies, the recording electrode was placed on the abductor hallucis, and the stimulation was applied 7.5 cm proximal to the recording site. For peroneal motor NCSs, the active electrode was placed on the extensor digitorum brevis, with distal stimulation 7 cm proximal to the active electrode. Sensory NCSs were performed antidromically. For sural sensory NCSs, the active electrode was placed posterior to the lateral malleolus, the reference electrode 3 cm distal, and stimulation was applied 12 cm proximal to the active electrode over the distal posterolateral leg. For SP sensory NCS, the active electrode was placed on the anterior part of the ankle, the reference electrode 3 cm distal, and stimulation was applied 12 cm proximal to the active electrode over the distal anterolateral leg.

Statistical analyses

Statistical analyses were conducted using the Statistical Package for the Social Sciences (SPSS) version 25.0 (Armonk, NY: IBM Corp.). Descriptive statistics were presented using numbers and percentages for categorical variables and mean±SD or median, range (minimum and maximum) for continuous variables. The Kolmogorov-Smirnov test was used to assess normality. After ensuring the normal distribution of the data with logarithmic transformation, side-to-side differences in CMAP and SNAP amplitudes were analyzed to determine upper limits, defined as mean+2SD. Given that the sural nerve originates from both the tibial and peroneal nerves, and the SP nerve only from the peroneal nerve, the relationships between associated motor and sensory nerves were evaluated using the Pearson correlation test. Where significant correlations were found between SNAP and CMAP amplitudes, linear regression was used to increase the predictive power of the covariation and to formulate this correlation. Significance was established at p<0.05.

## Results

NCSs were performed on 50 healthy adults (25 females, 25 males) aged 20-69 years with a mean of 38.52±14.3 years. Mean height was 170.16±6.11 cm and mean body mass index (BMI) 24.28±2.94 (Table 1).

**Table 1:** Demographic and anthropometric features of participants. Min: minimum; max: maximum

Variables	Female participants (n=25)	Male participants (n=25)	All participants (n=50)
Mean age (±SD)	30.68±7.23	46.36±15.41	38.52±14.3
Median age (min-max)	29 (21-48)	45 (20-69)	36 (20-69)
Mean body height (±SD) (cm)	165.24±4.72	175.08±1.91	170.16±6.11
Median body height (min-max) (cm)	164 (160-178)	175 (172-179)	172.5 (160-179)
Mean BMI (±SD)	24.21±3.53	24.34±2.28	24.28±2.94
Median BMI (min-max)	23.6 (18.2-30.9)	23.9 (19-30.3)	23.8 (18.2-30.9)

**Figure 1:**
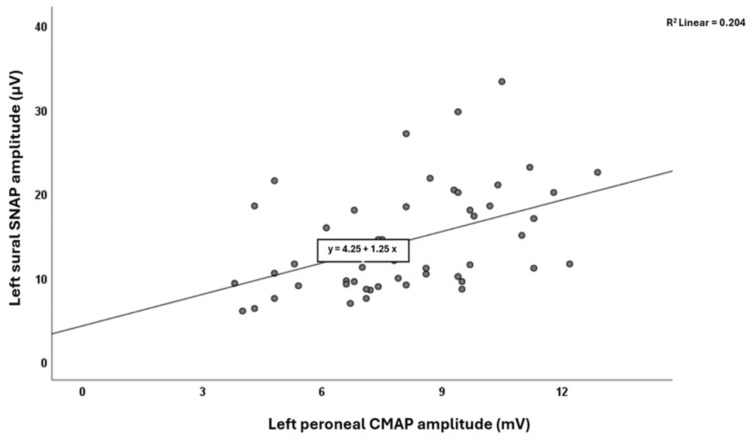
The correlation graph between left peroneal CMAP and left sural SNAP amplitudes. SNAP: sensory nerve action potential; CMAP: compound muscle action potential

**Figure 2:**
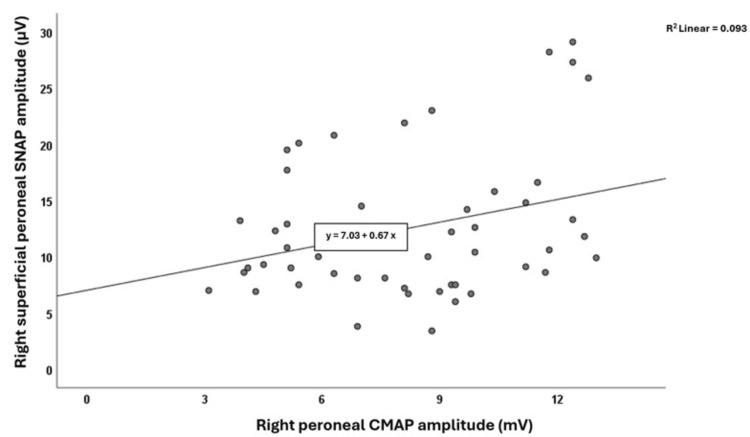
The correlation graph between right peroneal CMAP and right superficial peroneal SNAP amplitudes. SNAP: sensory nerve action potential; CMAP: compound muscle action potential

The electrophysiological findings for CMAP and SNAP amplitudes in both lower extremities are presented in Table 2. Statistically significant positive correlations were observed between left peroneal CMAP and left sural SNAP amplitudes (r=0.451, p=0.001) (Figure 1), and between right peroneal CMAP and right SP SNAP amplitudes (r=0.304, p=0.032) (Figure 2). Linear regression analyses showed that for the left sural SNAP amplitudes, the left peroneal CMAP amplitudes, and for the right SP SNAP amplitudes, the right peroneal CMAP amplitudes were found to be statistically significantly effective at B=1.246 (confidence interval: 0.531; 1.960, p=0.001) and B=0.672 (confidence interval: 0.062; 1.282, p=0.032), respectively. The upper limits for side-to-side differences (mean+2SD) were 7.76 µV for sural SNAP, 6.7 µV for SP SNAP, and 4.04 mV for peroneal CMAP amplitudes (Table 2). Only a few participants exceeded these cut-offs (n=2 for sural, n=4 for SP), and their CMAP amplitudes did not show a pattern of abnormality.

**Table 2:** Electrophysiological parameters and upper limits for side-to-side differences of participants. CMAP: compound muscle action potential; SNAP: sensory nerve action potential; min: minimum; max: maximum; N/A: not applicable

Variables	Tibial nerve		Peroneal nerve		Sural nerve		Superficial peroneal nerve	
	Right CMAP (mV)	Left CMAP (mV)	Right CMAP (mV)	Left CMAP (mV)	Right SNAP (µV)	Left SNAP (µV)	Right SNAP (µV)	Left SNAP (µV)
Mean of the amplitude (SD)	15.64 (5.39)	14.6 (4.93)	8.21 (2.88)	8.07 (2.29)	14.41 (6.56)	14.3 (6.34)	12.54 (6.37)	12.72 (7.25)
Median of the amplitude (min-max)	16.1 (6-26.5)	14.45 (6.5-28.6)	8.45 (3.1-13)	8 (3.8-12.9)	12.9 (5-33)	11.6 (6-33.3)	10.5 (3.4-29.1)	10.3 (3.3-33.8)
Upper limits of side-to-side difference (mV)	6.93		4.04		7.76		6.7	
Number of values beyond ±2SD	N/A		0		2		4	

When the condition of peroneal CMAP amplitude side-to-side difference being less than 4.04 mV (mean+2SD) is met, side-to-side differences of the sural and SP SNAP amplitudes up to 7.76 and 6.7 µV could be considered within normal limits. If peroneal CMAP amplitude asymmetry exceeds 4.04 mV, side-to-side SNAP comparisons may not follow the proposed normative limits, warranting individual review of the peroneal motor nerve conduction study technique and interpretation of the findings on an individual basis.

## Discussion

In this study, we kept the age and sex variables in line with the normal distribution, and the body height and BMI variables away from extreme values ​​and within certain ranges. We took into account that the sural nerve most commonly (80.7%) originates from both the peroneal and tibial nerves (type A formation), and that the SP sensory nerve originates from the peroneal nerve [6]. The upper limits of side-to-side differences in SNAP amplitudes were determined based solely on their association with lower extremity motor nerves. The relatively higher side-to-side differences in SNAP amplitudes in our study may be attributed to the limited number of older participants [7,8].

The sural nerve is known for its anatomical variability. Multiple cadaveric and electrophysiological studies have documented this in healthy adults [2,5,7,9]. Recent ultrasonographic research has also highlighted the anatomical variations of the sural nerve [10]. Similarly, the SP nerve may course through either the anterior or lateral compartments, as noted in the variants [4,11]. It is known that there are many variants of branching within the compartments and in the suprafascial layer during its course [4,12].

From a neurophysiological perspective, aside from other potential sources of side-to-side difference, including subcutaneous edema, averaging effects, and temperature differences, the anatomical variations of the sensory nerves may significantly contribute to discrepancies in NCSs between the two lower extremities [13]. Such variability may lead to incorrect electrophysiological diagnoses, including asymmetric polyneuropathy, plexopathy, and peripheral nerve injury. The variable course of the peroneal nerve is also critical in the context of entrapment syndrome. On the other hand, if technically suboptimal recordings of the sural and SP sensory nerves are performed, asymmetric findings and misdiagnoses may be encountered. NCSs should be performed with utmost technical care, considering the possibility of anatomical variation of the nerve. Regarding the sural nerve, some studies have been conducted suggesting different neurophysiological methods that may contribute to the side-to-side difference issue. Considering the variability of the medial and lateral sural cutaneous nerve anastomosis of the sural nerve, Pyun and Kwon determined five different stimulation points in a horizontal line and obtained a double-peaked sural SNAP with stimulation from a more distal point in their study [14]. Tankişi et al. stated that in suspicion of the variation, different electrode placements could be considered in their study evaluating the anatomical variation of the sural nerve with both surface electrodes and near-nerve technique [2]. Winkel et al. focused on optimizing existing methods for SP sensory NCS asymmetry [15]. However, no study has yet proposed a novel neurophysiological approach specifically for addressing SP SNAP asymmetry.

Limitations

The sample size of 50 participants may be at the lower limit for conducting robust statistical analyses. However, this study serves as a pilot investigation, with the methodology intended to inform and guide future large-scale research. The observation that a statistically significant correlation with peroneal CMAP amplitude was found only between the left sural and the right SP SNAP amplitudes may reflect limited statistical power of the study and/or inter-individual variability, rather than true biological asymmetry. Another limitation is the potential presence of skewed distributions in biological data. Given that biological variability may include outliers or non-normal distributions, future studies may benefit from employing non-parametric or percentile-based statistical approaches.

## Conclusions

Based on current literature and our findings, side-to-side differences in sural and SP SNAP amplitudes may be present even under technically optimal conditions. Considering the possibility of any anatomical variation, these differences are accepted within the normal range up to amplitude differences of roughly 50%. To draw attention to the risk of misdiagnosis in electrodiagnostic evaluations, we precisely calculated the upper limits of the side-to-side sural and SP SNAP amplitude differences and suggested considering the peroneal CMAP amplitude, which may affect these differences, as an interpretive guide for the side-to-side SNAP amplitude difference evaluation. Future studies with larger samples and using certain types of electrodes could support our findings for both sides and further suggest that the sural nerve may be associated with both the tibial and peroneal nerves. We hope that our study may serve as a basis for further studies with larger samples that include age range groupings and use the methodology of our study to determine the upper limit values of the side-to-side sural and SP SNAP amplitude differences, preferably conducted in multiple centers to ensure the technical factors.
